# A pragmatic approach to the design and calibration of a Bayesian CRM dose finding trial

**DOI:** 10.1186/1745-6215-16-S2-P210

**Published:** 2015-11-16

**Authors:** Michael Cole, Deborah Stocken, Christina Yap

**Affiliations:** 1 Newcastle University, Newcastle upon Tyne, UK; 2 University of Birmingham, Birmingham, UK

## 

The Continual Reassessment Method (CRM) is not widely used in early phase dose finding trials partly because the logistics of development and implementation are perceived as complex and time consuming. Details are presented of the steps involved when designing such a trial using an adaptation of a Bayesian CRM calibration algorithm proposed by Cheung [1].

In addition to clinician centred parameters including target toxicity probability and number of test doses, the Bayesian CRM requires specification of the prior probability of toxicity associated with each of the test doses and the prior SD of the single model parameter. Cheung’s simple pragmatic approach is used to aid selection of these parameters.

Competing designs are assessed in terms of risk-adjusted accuracy and trial specific requirements including the mean number of patients treated above the maximum tolerated dose. Performance measures are estimated by Monte Carlo simulation across a range of scenarios chosen solely upon the target toxicity level and the number of test doses.

We demonstrate that following this pragmatic approach to CRM design calibration, design of CRM trials need not be overly complex or time-consuming.
